# Association of paraspinal fat infiltration with postoperative clinical outcomes in older cervical spondylotic myelopathy patients undergoing anterior cervical discectomy and fusion

**DOI:** 10.1186/s12877-026-08026-0

**Published:** 2026-07-24

**Authors:** Yan Gong, Hao Liu, Yuhong Zhang, Suliang Xu, Yuyang Shao, Shun Lin, Zhiqiang Wang, Hang Zhuo, De Liang, Zhensong Yao, Xiaochun Li, Jintao Liu

**Affiliations:** 1 Suzhou TCM Hospital Affiliated to Nanjing University of Chinese Medicine, Suzhou, 215009 China; 2 Nanjing University of Chinese Medicine, Nanjing, 210023 China; 3 The First Affiliated Hospital of Guangzhou University of Chinese Medicine, Guangzhou, 510405 China

**Keywords:** Fat infiltration, Older patients with cervical spondylotic myelopathy, Anterior cervical discectomy and fusion, Goutallier classification, Clinical outcomes

## Abstract

**Objective:**

To investigate the association between preoperative cervical paraspinal muscle fat infiltration and 2‑year clinical outcomes in older patients with cervical spondylotic myelopathy (CSM) undergoing anterior cervical discectomy and fusion (ACDF), and to analyze its relationship with postoperative radiographic correction parameters and long‑term pain prognosis.

**Methods:**

A retrospective analysis was conducted on 91 older CSM patients who underwent ACDF between January 2020 and June 2023. Based on preoperative MRI assessment using the Goutallier classification system, patients were stratified into three groups according to the degree of paraspinal muscle fat infiltration: mild (Grade 0–1, *n* = 36), moderate (Grade 1.5–2, *n* = 31), and severe (Grade 2.5–4, *n* = 24). Baseline data, key sagittal radiographic parameters, and clinical outcome scores (JOA, NDI, VAS) were compared among groups. Multiple linear regression analysis was performed to identify independent factors associated with the VAS score at 2-year follow-up.

**Results:**

Postoperative radiographic parameters, including the T1 slope, C2 slope, C2–C7 Cobb angle, C0–C2 Cobb angle, sagittal segmental angle (SSA), and average segmental disc height (ASDH), significantly improved in all patients (*P* < 0.05). Clinical scores (JOA, NDI, VAS) also showed significant improvement at the 2-year follow-up (*P* < 0.05). However, the severe fat infiltration group demonstrated significantly worse immediate postoperative VAS scores, as well as worse NDI and VAS scores at the 2-year follow-up compared to the mild and moderate groups (*P* < 0.05). Multiple linear regression analysis confirmed that the preoperative Goutallier grade was a significant independent factor associated with a higher VAS score at 2 years (B = 0.962, *P* < 0.001), after adjusting for relevant covariates.

**Conclusion:**

ACDF effectively improves clinical symptoms and cervical alignment in older CSM patients. However, severe preoperative paraspinal muscle fat infiltration is independently associated with inferior postoperative pain relief and functional recovery. Assessment of paraspinal muscle status may be useful for risk stratification in older patients undergoing ACDF.

## Introduction

As the global population continues to age at an accelerating pace, the incidence of degenerative cervical diseases has been steadily increasing, becoming a significant health issue that impacts the quality of life of middle-aged and older individuals [1]. Among these, cervical spondylotic myelopathy (CSM) represents one of the most severe forms of cervical spondylosis. It arises from factors such as cervical disc degeneration, ligament hypertrophy, and osteophyte formation, which compress or compromise the blood supply to the spinal cord. This leads to sensory, motor, and sphincter dysfunction in the limbs [2]. When a patient’s neurological function progressively deteriorates and systematic conservative treatment is ineffective, surgical intervention becomes the key approach to relieve spinal cord compression and halt disease progression [3]. Anterior cervical discectomy and fusion (ACDF) has become the standard surgical approach for treating CSM because of its ability to directly remove compressive lesions, restore intervertebral disc height, and achieve stable intervertebral fusion [4]. For patients with CSM undergoing ACDF, the surgical goal extends beyond effective decompression to include the restoration and maintenance of normal sagittal plane alignment of the cervical spine. This indicator has been demonstrated in multiple studies to be closely associated with postoperative functional recovery and long-term efficacy [5, 6].

In recent years, with a deeper understanding of cervical spine biomechanics, the research focus has expanded from purely bony structures to the balance of surrounding soft tissues, particularly the structural and functional states of paravertebral muscle groups [7, 8]. The paravertebral muscles of the cervical spine, as the core structures responsible for maintaining dynamic stability, enabling neck movement, and supporting the head’s weight, have increasingly drawn academic attention because of their degenerative changes [9]. Among the various forms of degeneration, muscle-fat infiltration has become a key radiographic indicator for assessing the quality and function of paraspinal muscles [10]. Research indicates that fat infiltration of the paraspinal muscles in the lumbar spine is significantly associated with the occurrence of lumbar degenerative diseases, the severity of clinical symptoms, and postoperative outcomes [11, 12]. Similarly, fat infiltration of the cervical paraspinal muscles has also been demonstrated to be closely associated with sagittal plane imbalance of the cervical spine, chronic neck pain, whiplash injury sequelae, and postoperative axial symptoms [13–15]. Fat infiltration not only reflects a decrease in muscle function but also may alter the biomechanical environment of the cervical spine and weaken its dynamic stability, thereby affecting postoperative recovery and clinical outcomes [16].

Currently, the Goutallier grading system is a qualitative visual assessment method based on MRI evaluation of paraspinal muscle fat infiltration, demonstrating good clinical applicability [17]. Therefore, conducting an in-depth investigation into the role of paraspinal muscle fat infiltration on the clinical outcomes of patients with cervical spondylotic myelopathy and ACDF surgery holds clear clinical significance. However, the extent to which preoperative fat infiltration affects mid- to long-term functional recovery after ACDF in patients with CSM remains to be further clarified. Therefore, this study aimed to systematically investigate the impact of preoperative cervical paraspinal muscle fat infiltration, stratified according to the Goutallier classification, on 2-year postoperative clinical efficacy and functional scores in patients with cervical spondylotic myelopathy who underwent ACDF. Through retrospective analysis of this study, we aimed to provide evidence-based support for identifying high-risk patients with potential poor postoperative recovery, optimizing their perioperative assessment and management strategies, and thereby advancing the precision and individualization of CSM surgical treatment.

## Materials and methods

### Participants

This single-center retrospective analysis consecutively enrolled 91 patients who underwent ACDF surgery at the First Affiliated Hospital of Guangzhou University of Chinese Medicine between January 2020 and June 2023 for short-segment (≤ 2 segments involved) cervical spondylotic myelopathy. All patients underwent standardized postoperative follow-up for 2 years. The inclusion criteria included the following: (1) Age 60 years or older; (2) A definitive diagnosis of CSM based on characteristic clinical symptoms (e.g., sensory or motor deficits in the limbs, gait instability) and corroborative cervical MRI findings, with involvement of no more than two spinal segments; (3) Inadequate improvement or persistent progression of symptoms after a minimum of 3 months of systematic conservative treatment (e.g., physical therapy, analgesic medication), with neurological impairment impacting activities of daily living; (4) Candidates for primary ACDF surgery with complete preoperative imaging (including cervical X-ray and MRI) available for assessment.The exclusion criteria were as follows: (1) history of cervical spine surgery; (2) history of trauma that may alter the cervical spine structure, such as cervical fracture or dislocation; (3) long-term use of antiplatelet or anticoagulant medications (e.g., aspirin, clopidogrel) with failure to complete standardized preoperative discontinuation as needed, resulting in a high risk of perioperative bleeding; (4) severe systemic comorbidities, such as cardiovascular, cerebrovascular, hepatic, or renal insufficiency, classified as high-risk surgical candidates; and (5) loss to follow-up or incomplete follow-up data, precluding effective efficacy assessment. This study protocol was approved by the Ethics Review Committee of the First Affiliated Hospital of Guangzhou University of Chinese Medicine (Approval No. K2020104). All enrolled patients signed informed consent forms specific to this study prior to surgery.

All patients were stratified into three groups based on the preoperative Goutallier grade, which was calculated as the mean of the right and left paraspinal muscle assessments, allowing for half-grades (e.g., 1.5, 2.5). Accordingly, patients were classified as: mild (Grade 0–1, i.e., mean grade ≤ 1, *n* = 36), moderate (Grade 1.5–2, i.e., mean grade > 1 to ≤ 2, *n* = 31), and severe (Grade 2.5–4, i.e., mean grade > 2, *n* = 24). This classification has been previously used in studies evaluating cervical paraspinal muscles [15], and its reliability in our cervical cohort was confirmed by an inter-rater weighted κ of 0.85.

### Surgical procedure

All patients who met the inclusion criteria underwent ACDF surgery under general anesthesia performed by the same senior spine surgeon. Patients were placed in the supine position with a transverse cervical incision. Layers were dissected sequentially via the platysma and deep fascial approach. The carotid sheath was retracted laterally, and the trachea and esophagus were retracted medially to fully expose the prevertebral structures. Under C-arm fluoroscopic guidance, the affected segment was precisely localized. The annulus fibrosus was then incised, and the nucleus pulposus tissue was completely removed. Standardized processing of the intervertebral space and cartilaginous endplates was performed. The Caspar intervertebral retractor was inserted and secured firmly. The posterior longitudinal ligament was incised, adhesions were carefully dissected, and the spinal canal was explored. All disc material protruding into the canal was thoroughly removed, and constant care was taken to protect the spinal cord from injury throughout the procedure. The posterior vertebral elements were further decompressed, and the uncovertebral joints were exposed to achieve adequate decompression. A properly sized interbody fusion cage was selected, filled with a bone graft substitute, and securely implanted into the intervertebral space. The retractor was removed, a titanium plate was placed on the anterior vertebral body, and its stability was verified. The satisfactory position of the internal fixation was confirmed via C-arm fluoroscopy. The surgical site was routinely irrigated, a drainage tube was placed, and the incision was closed in layers. Postoperatively, the patient received vital sign monitoring and standard prophylactic antibiotics for 24 h. The drainage tube was removed 24–48 h postoperatively. The patient was instructed to wear a cervical collar for 8–12 weeks and gradually initiate neck functional exercises under medical supervision, tailored to individual recovery progress.

### Radiographic analyses and data collection

**Fig. 1:**
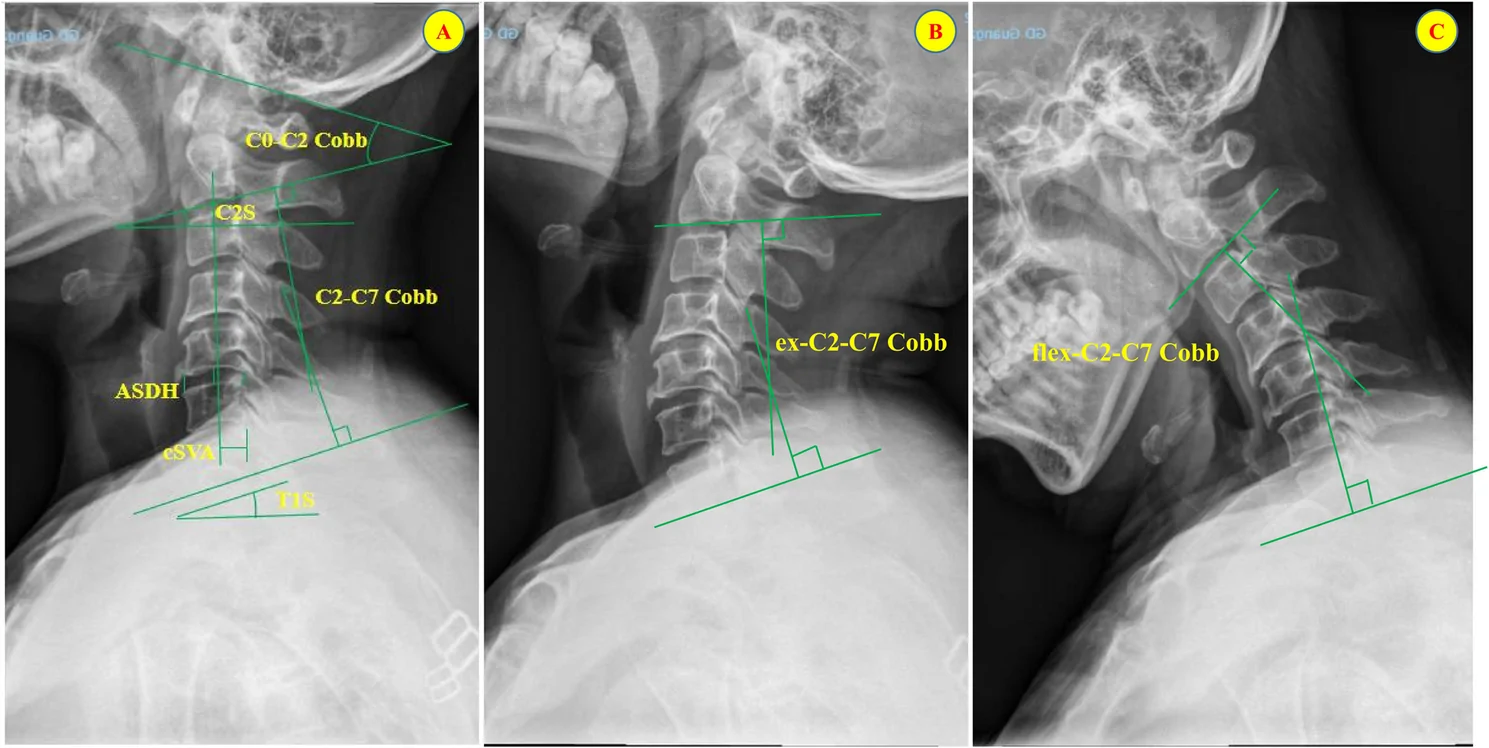
Measurement of cervical sagittal parameters. **A**: Neutral lateral radiograph demonstrating the C2–C7 Cobb angle, C0–C2 Cobb angle, cSVA, ASDH, T1 slope, and C2 slope; **B**:Hyperextension view showing the C2–C7 Cobb angle; **C**: Hyperflexion view showing the C2–C7 Cobb angle

This study systematically collected baseline data and perioperative indicators for all enrolled patients, including age, sex, duration of surgery, intraoperative blood loss, and BMI. Preoperative and postoperative lateral and dynamic X-ray images of the cervical spine were acquired to assess the following sagittal plane parameters: the C2–C7 Cobb angle, defined as the angle formed between the vertical line connecting the inferior endplates of C2 and C7; the C0–C2 Cobb angle, defined as the angle between McRae’s line and the inferior endplate of C2; the C2–7 sagittal vertical axis (cSVA), defined as the vertical distance from the center of the C2 vertebral body to the posterior superior angle of the C7 vertebral body; the T1 slope, defined as the angle between the horizontal line and the line connecting the superior endplates of the T1 vertebral body; and the C2 slope, defined as the angle between the horizontal line and the line connecting the inferior endplates of the C2 vertebral body. The surgical segmental angle (SSA) measures the angle formed between the superior endplate of the superior vertebra and the inferior endplate of the inferior vertebra within the surgical segment. The average segmental disc height (ASDH) is the arithmetic mean of the anterior, middle, and posterior third heights of the intervertebral space within that segment. Additionally, on the basis of dynamic cervical spine imaging, the range of motion (ROM) of the vertebral bodies above and below the surgical segment (upper/lower-SSA-ROM) is further extracted. The specific measurement methods for each parameter are detailed in Fig. 1. All radiographic measurements and Goutallier grading were performed independently by two senior spine surgeons who were blinded to the patients’ clinical data, including Japanese Orthopaedic Association (JOA), neck disability index (NDI), and visual analog scale (VAS) scores, as well as to each other’s measurements. The inter-rater reliability for all radiographic measurements was assessed using the intraclass correlation coefficient (ICC, two-way random effects model for absolute agreement), with ICC values exceeding 0.90 indicating excellent agreement.

Postoperative recovery was assessed by the JOA, VAS and NDI scores, which were collected preoperatively, immediately after surgery, and at the 2-year follow-up. The VAS was used to assess the patient’s overall pain intensity (including neck, axial, and referred pain), without specific differentiation between neck pain and arm pain. All scoring results were jointly verified and evaluated by two senior clinicians.

**Fig. 2:**
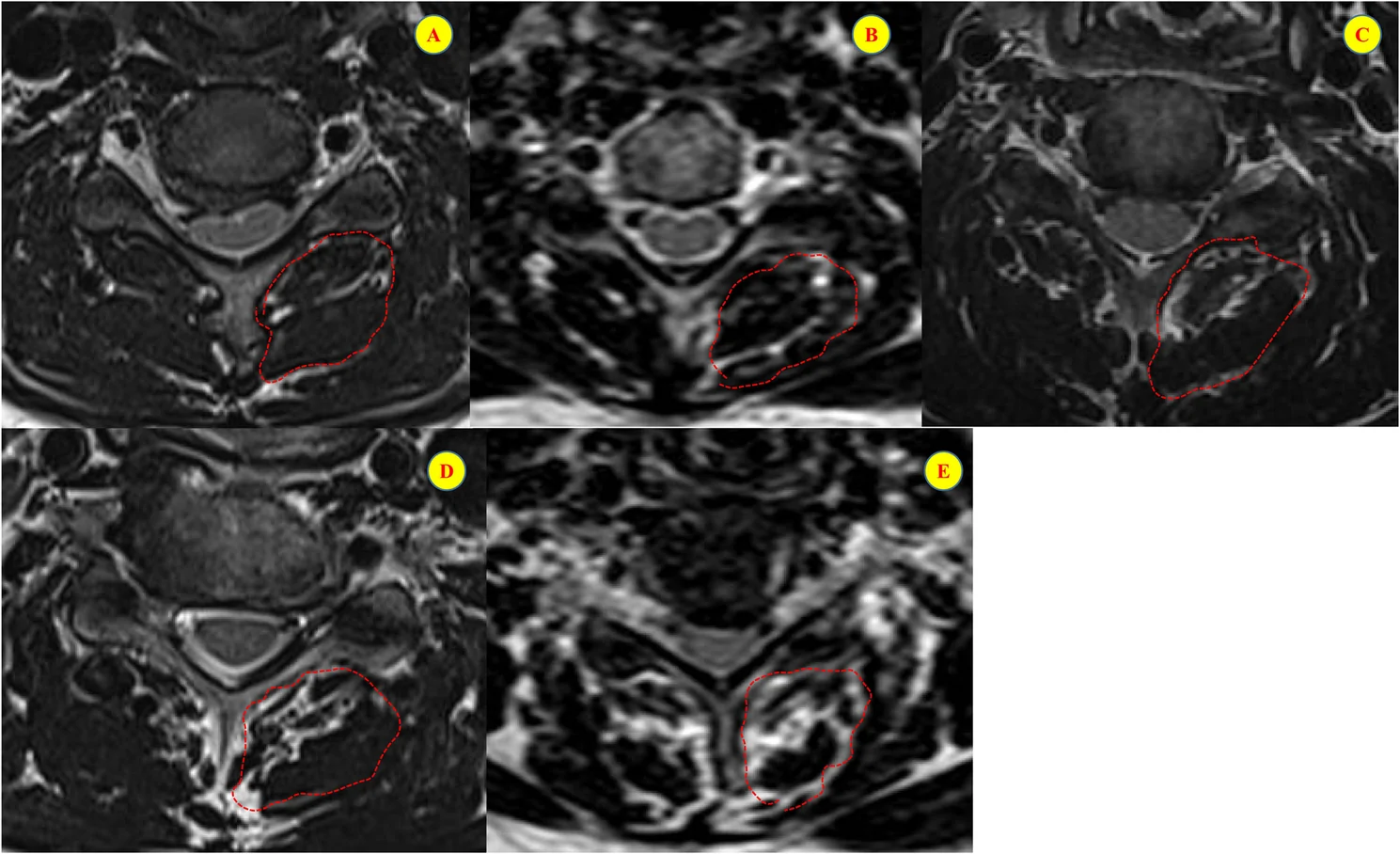
T2-weighted axial images obtained at the C5–6 intervertebral level, used to assess and grade paraspinal muscle fat infiltration. Panels (**A**), (**B**), (**C**), (**D**), and (**E**) correspond to grades 0, I, II, III, and IV fat infiltration, respectively

Additionally, T2-weighted MRI at the C5-6 segment level was collected from all patients preoperatively to assess paraspinal muscle function status. The C5–6 level was selected because it is the most commonly affected and surgically treated segment in cervical spondylotic myelopathy, providing a consistent and reproducible anatomical landmark for measuring the cervical paraspinal muscles. This approach is consistent with previous cervical muscle imaging studies [18]. The degree of fat infiltration in the cervical paraspinal muscles (including the multifidus and semispinalis cervicis) was evaluated using the Goutallier grading system [8], a qualitative visual assessment framework with scores ranging from 0 to 4. This system, originally developed for the lumbar spine, has been widely applied in cervical spine research and its reliability in cervical assessment has been demonstrated. In our cohort, the inter-rater reliability for Goutallier grading was excellent (weighted κ = 0.85). The right and left multifidus muscles were assessed separately, with the final classification determined by averaging the scores from both sides. Based on this average, Goutallier grades were defined as follows: Grade 0, no visible fat striations; Grade I, minimal fat striations; Grade II, muscle content exceeding fat content; Grade III, fat and muscle content roughly equal; and Grade IV, fat content exceeding muscle content. This grading system was jointly verified and determined by two senior spine surgeons. See Fig. 2 for a schematic illustration.

### Statistical analysis

All statistical analyses were performed using SPSS 27.0 software. Continuous variables are expressed as means ± standard deviations, and categorical variables as frequencies or percentages. The normality of distribution for all continuous variables was assessed using the Shapiro–Wilk test. Comparisons of categorical variables between groups were performed using the chi‑square test. For continuous variables, one‑way ANOVA was used for intergroup comparisons when data met normality assumptions; otherwise, the Kruskal–Wallis test was applied. When the overall ANOVA was significant or showed a borderline trend (*P* < 0.10), post hoc pairwise comparisons were performed using the least significant difference (LSD) method for normally distributed data; for nonnormally distributed data, Dunn's test with Bonferroni correction was used. Differences between preoperative and postoperative measurements within the same patient were compared using paired t‑tests (for normally distributed data) or Wilcoxon signed‑rank tests (for nonnormally distributed data). Statistical significance was set at *P* < 0.05 for all tests.

## Results

### Patient demographics

The inter-rater reliability for Goutallier grading was excellent (weighted κ = 0.85). The ICCs for all radiographic measurements exceeded 0.90, indicating excellent agreement. This study included a total of 91 patients with CSM, comprising 39 males and 52 females. According to the Goutallier classification system, all patients were divided into three groups: the mild group (grade 0–1), comprising 36 patients with a mean age of 65.97 ± 4.88 years; the moderate group (grade 1.5–2), comprising 31 patients with a mean age of 65.90 ± 5.35 years; and the severe group (grade 2.5–4.0), comprising 24 patients with a mean age of 64.92 ± 3.91 years. There were no statistically significant differences among the three patient groups in terms of baseline characteristics, including age, sex, BMI, duration of surgery, intraoperative blood loss, ROM, or upper/lower SSA-ROM (*P* > 0.05). The distribution of surgical segments (single-level vs. two-level) and operated levels (C3–4 to C6–7) was balanced across the three groups (all *P* > 0.05, Table 1).

**Table 1:** Baseline data among the three groups

Parameters	Mild group(0–1)	Moderate group(1.5-2.0)	Severe group(2.5-4.0)	*P* value
No	36	31	24	
Age(years)	65.97 ± 4.88	65.90 ± 5.35	64.92 ± 3.91	0.672
Sex(male/female)	13/23	13/18	13/11	0.380
Surgical segments, n (%)				0.979
Single-level	24 (66.7%)	20 (64.5%)	16 (66.7%)	
Two-level	12 (33.3%)	11 (35.5%)	8 (33.3%)	
Operated segments, n (%)				1.000
C3–4	6 (12.5%)	5 (11.9%)	4 (12.5%)	
C4–5	12 (25.0%)	11 (26.2%)	8 (25.0%)	
C5–6	19 (39.6%)	16 (38.1%)	13 (40.6%)	
C6–7	11 (22.9%)	10 (23.8%)	7 (21.9%)	
BMI(kg/m²)	23.22 ± 2.81	23.18 ± 3.33	23.73 ± 2.68	0.757
Operation time (min)	127.50 ± 31.27	127.90 ± 40.67	132.54 ± 31.40	0.841
Blood loss (mL)	31.94 ± 32.32	29.68 ± 22.13	37.08 ± 23.68	0.595
ROM(°)	28.66 ± 16.55	34.32 ± 16.16	32.04 ± 17.98	0.385
Upper-SSA-ROM(°)	7.17 ± 3.61	9.44 ± 6.48	9.72 ± 5.74	0.160
Lower-SSA-ROM(°)	8.60 ± 4.84	11.34 ± 5.40	9.11 ± 4.80	0.074

### Comparison of cervical parameters & efficacy scores: pre- vs. postoperative in all patients

**Fig. 3:**
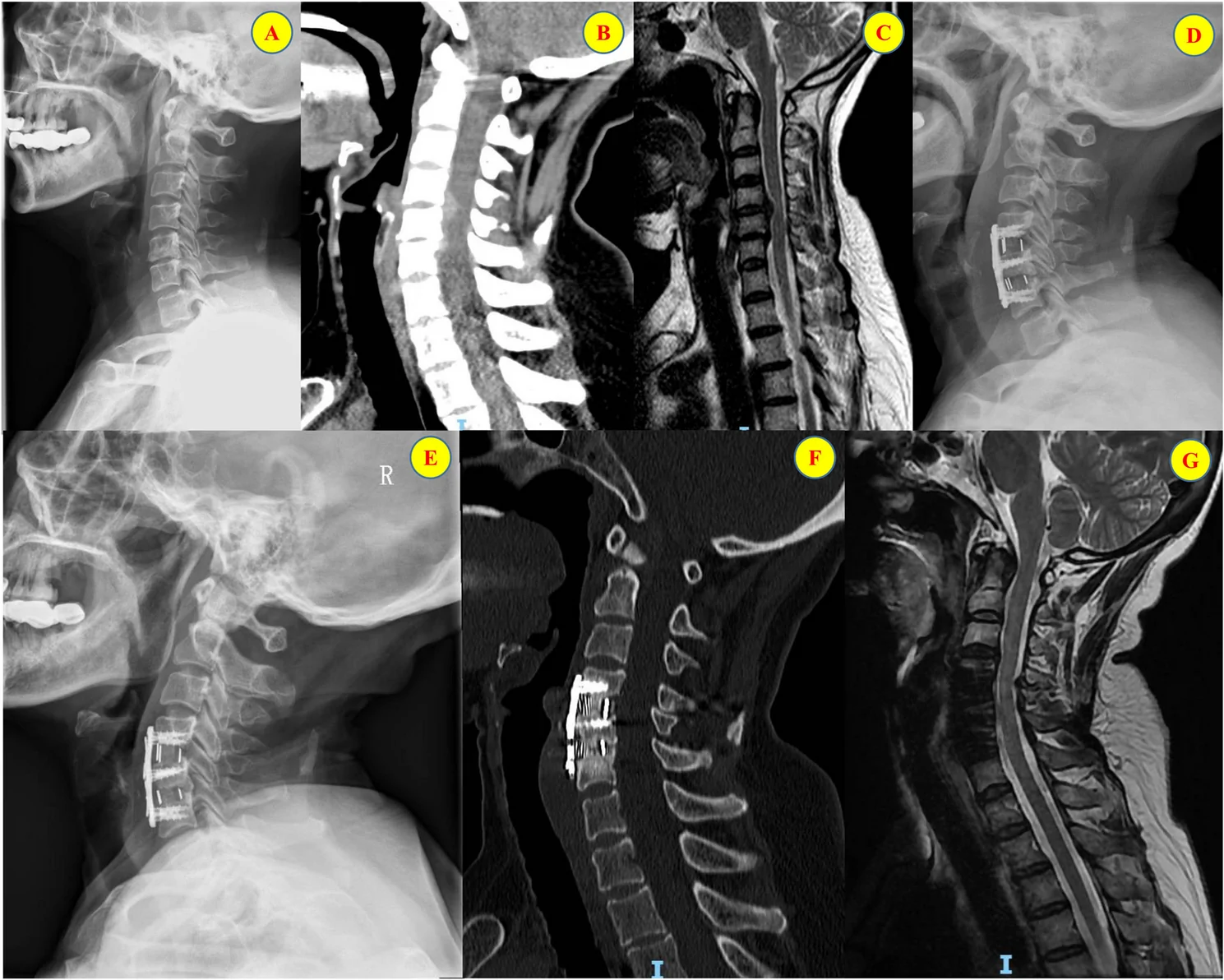
A 62-year-old male patient underwent ACDF for C4‒C6 segmental pathology. Preoperative imaging (**A**-**C**) revealed disc herniation at the C4/5 and C5/6 levels with corresponding segmental spinal canal stenosis, marked spinal cord compression at C4/5, and loss of cervical lordosis. Immediate postoperative radiographs (**D**) demonstrated good positioning of the internal fixation. Follow-up imaging at 2 years (**E**-**G**) further confirmed stable internal fixation placement, good restoration of the cervical sagittal alignment, and bony fusion at the operative level, indicating durable and stable surgical outcomes

Postoperative imaging assessments of all patients in this study demonstrated significant improvements in the T1 slope, C2‒C7 Cobb angle, C0‒C2 Cobb angle, C2 slope, SSA, and ASDH compared with the preoperative measurements (*P* < 0.05, Table 2). Compared with the preoperative values, the JOA, NDI, and VAS scores at the 2-year postoperative follow-up also markedly improved (*P* < 0.05, Table 3). These findings confirm that ACDF surgery is highly effective in alleviating clinical symptoms and restoring and maintaining cervical sagittal sequence balance. A typical follow-up case is shown in Fig. 3.

**Table 2:** Comparison of preoperative and postoperative parameters for all patients

Parameters(*n* = 91)	Preoperative	Postoperative	*P* value
T1 slope(°)	22.03 ± 7.17	27.19 ± 7.26	< 0.001
C2-C7 Cobb(°)	9.34 ± 5.71	17.29 ± 7.25	< 0.001
SSA(°)	5.92 ± 4.31	10.91 ± 4.90	< 0.001
C0-C2 Cobb(°)	32.08 ± 6.49	23.34 ± 6.17	< 0.001
cSVA(mm)	21.53 ± 11.50	21.04 ± 11.05	0.678
C2 slope (°)	8.17 ± 4.81	10.56 ± 6.01	0.001
ASDH(mm)	5.78 ± 1.18	8.97 ± 1.29	< 0.001

**Table 3:** All patients were compared at their final follow-up visit with their preoperative scores

Parameters(*n* = 91)	Preoperative	Final follow-up	*P* value
JOA (score)	12.38 ± 2.48	15.26 ± 1.26	< 0.001
NDI (score)	22.92 ± 8.69	4.00 ± 2.39	< 0.001
VAS (score)	4.98 ± 1.63	1.89 ± 1.43	< 0.001

### Intergroup comparison of cervical parameters, scores, and score improvement among the three groups

The results of this study indicate that there were no statistically significant differences among the three groups in terms of the T1 slope, C2‒C7 Cobb angle, C0‒C2 Cobb angle, C2 slope, SSA, or ASDH (*P* > 0.05, Table 4). Regarding clinical scores, no significant differences were observed among the three groups in preoperative JOA, immediate postoperative JOA, 2‑year follow‑up JOA, preoperative NDI, immediate postoperative NDI, or preoperative VAS (*P* > 0.05). However, significant differences were observed among the three groups in NDI and VAS at 2‑year follow‑up, as well as in immediate postoperative VAS (*P* < 0.05).

**Table 4:** Comparison of preoperative and postoperative parameters among the three groups

Parameters	Mild group(0–1)	Moderate group(1.5-2.0)	Severe group(2.5-4.0)	*P* value
C2-C7cobb(°)				
Preoperative	8.68 ± 4.47	8.73 ± 6.30	11.12 ± 6.39	0.207
Postoperative	17.39 ± 6.90	16.26 ± 7.72	18.75 ± 7.32	0.533
C0-C2cobb(°)				
Preoperative	31.91 ± 5.59	33.72 ± 6.54	30.20 ± 7.35	0.134
Postoperative	23.39 ± 6.35	24.57 ± 5.85	21.66 ± 6.17	0.222
cSVA(mm)				
Preoperative	23.22 ± 11.51	20.34 ± 11.28	20.51 ± 11.49	0.527
Postoperative	19.98 ± 11.02	20.43 ± 10.04	23.40 ± 12.38	0.471
T1 slope (°)				
Preoperative	21.82 ± 6.98	21.60 ± 7.72	22.97 ± 6.92	0.753
Postoperative	27.23 ± 6.26	26.17 ± 7.98	28.47 ± 7.72	0.515
C2 slope (°)				
Preoperative	9.03 ± 5.71	8.43 ± 3.74	6.57 ± 4.53	0.143
Postoperative	9.96 ± 5.91	10.70 ± 6.04	11.26 ± 6.31	0.706
SSA(°)				
Preoperative	5.24 ± 3.79	5.91 ± 4.13	6.95 ± 5.16	0.323
Postoperative	10.47 ± 5.33	10.44 ± 3.90	12.17 ± 5.35	0.345
ASDH(mm)				
Preoperative	5.62 ± 1.20	5.88 ± 1.19	5.91 ± 1.22	0.563
Postoperative	8.93 ± 1.19	8.90 ± 1.53	9.05 ± 1.15	0.919

Further analysis revealed that, compared with the severe group, both the mild and moderate groups had significantly better outcomes, as evidenced by lower NDI and VAS scores at 2‑year follow‑up and lower immediate postoperative VAS (*P* < 0.05, Table 5). Regarding the degree of improvement, both the mild and moderate groups showed significantly greater VAS reduction than the severe group (*P* < 0.05). However, no significant difference was observed in JOA recovery rate among the three groups (*P* = 0.912, Table 6). For NDI improvement, the overall ANOVA showed a borderline trend (*P* = 0.059). Post hoc pairwise comparisons using the LSD method suggested a potential difference between the moderate and severe groups (*P* = 0.024), while the mild vs. severe difference approached but did not reach statistical significance (*P* = 0.053). These post hoc findings should be interpreted as exploratory given the borderline overall ANOVA result (Table 6).

**Table 5:** Comparison of preoperative, postoperative, and 2‑year follow‑up scores among the three groups

Parameters	Mild group(0–1)	Moderate group(1.5-2.0)	Severe group(2.5-4.0)	*P* value
JOA (score)				
Preoperative	12.36 ± 3.25	12.65 ± 2.12	12.08 ± 1.38	0.709
Postoperative	14.53 ± 1.16	14.29 ± 1.30	14.21 ± 1.35	0.584
Final follow-up	15.22 ± 1.51	15.45 ± 1.29	15.08 ± 0.72	0.550
NDI (score)				
Preoperative	23.06 ± 8.86	24.19 ± 9.97	21.08 ± 6.36	0.422
Postoperative	14.14 ± 5.75	14.52 ± 5.69	14.75 ± 4.97	0.911
Final follow-up	3.22 ± 2.03	3.39 ± 1.89	5.96 ± 2.42	< 0.001
VAS (score)				
Preoperative	4.75 ± 1.54	5.35 ± 1.47	4.83 ± 1.90	0.281
Postoperative	2.56 ± 1.65	2.68 ± 1.45	3.88 ± 0.74	0.001
Final follow-up	1.31 ± 1.28	1.42 ± 1.18	3.38 ± 0.71	< 0.001

**Table 6:** Comparison of clinical improvement among the three groups

Parameters	Mild group(0–1)	Moderate group(1.5-2.0)	Severe group(2.5-4.0)	*P* value
JOA recovery rate (%)*	52.69 ± 47.36	52.31 ± 50.69	56.93 ± 21.11	0.912
∆NDI (score)†	19.83 ± 8.57	20.81 ± 10.60	15.13 ± 7.69	0.059
∆VAS (score)†	3.44 ± 1.86	3.94 ± 2.10	1.46 ± 2.15	< 0.001

*JOA recovery rate was calculated using the Hirabayashi method: (postoperative JOA − preoperative JOA) / (17 − preoperative JOA) × 100 (%). †ΔNDI and ΔVAS were calculated as preoperative score minus final follow-up score, as lower scores indicate better function for these measures

### Comparison of radiographic correction magnitudes among groups

To evaluate the potential confounding effect of differential surgical correction, we compared the Δ values (postoperative minus preoperative) of key sagittal parameters among the three groups (Table 7). One-way ANOVA revealed no statistically significant differences among the groups for any of the parameters assessed, including the primary angles of cervical lordosis (ΔC2-C7 Cobb, ΔT1 slope), segmental alignment (ΔSSA, ΔASDH), as well as ΔcSVA and ΔC2 slope (all *P* > 0.05, Table 7).

**Table 7:** Comparison of the magnitude of correction (Δ value) in radiographic parameters among the three groups (postoperative value minus preoperative value)

Parameters	Mild group(0–1)	Moderate group(1.5-2.0)	Severe group(2.5-4.0)	*P* value
ΔcSVA (mm)	-3.24 ± 12.26	0.09 ± 11.16	2.89 ± 8.90	0.110
ΔT1 slope (°)	5.41 ± 6.90	4.61 ± 5.99	5.49 ± 5.22	0.831
ΔC0–C2 Cobb (°)	-8.53 ± 6.47	-9.15 ± 5.61	-8.54 ± 4.69	0.891
ΔC2–C7 Cobb (°)	8.72 ± 6.46	7.53 ± 6.54	7.36 ± 6.78	0.668
ΔC2 slope (°)	0.93 ± 7.55	2.28 ± 5.90	4.69 ± 6.14	0.105
ΔSSA (°)	5.24 ± 4.83	4.54 ± 3.57	5.22 ± 5.20	0.789
ΔASDH (mm)	3.31 ± 1.16	3.03 ± 1.30	3.14 ± 1.57	0.592

To further assess the relationship between the severity of preoperative fat infiltration and the magnitude of surgical correction, Spearman correlation analysis was conducted. The analysis revealed that a higher Goutallier grade was significantly correlated with greater postoperative increases in both ΔcSVA and ΔC2 slope (ρ = 0.221, *P* = 0.035 and ρ = 0.220, *P* = 0.036, respectively). In contrast, no significant correlations were observed with the Δ values of the primary parameters of cervical lordosis restoration (ΔC2‒C7 Cobb, ΔT1 slope) or segmental alignment (ΔSSA, ΔASDH) (all *P* > 0.05).

### Factors associated with 2-year postoperative VAS score: multiple linear regression analysis

A multiple linear regression analysis was performed to definitively assess whether preoperative paraspinal muscle fat infiltration is independently associated with long-term postoperative pain, after controlling for the magnitude of radiographic correction and other potential confounders. The dependent variable was the 2-year postoperative VAS score. The model was statistically significant (F(8, 82) = 5.357, *P* < 0.001), explaining 34.3% of the variance in the outcome (R² = 0.343, Adjusted R² = 0.279). The key results are presented in Table 8. The Goutallier grade of fat infiltration emerged as a significant independent factor associated with worse postoperative pain (unstandardized coefficient B = 0.962, 95% CI: 0.630 to 1.293; standardized coefficient β = 0.544; *P* < 0.001). This indicates that for each increment in the Goutallier grade, the 2-year postoperative VAS score increased by an average of 0.962 points, after accounting for all other variables in the model.

**Table 8:** Multiple linear regression analysis of factors associated with the 2-year postoperative VAS score

Variable	B (95% CI)	SE	β	t	*P* value
Constant	-1.293 (-5.820, 3.234)	2.285	—	-0.566	0.573
Goutallier grade	0.962 (0.630, 1.293)	0.167	0.544	5.763	< 0.001
ΔcSVA (mm)	0.019 (-0.007, 0.045)	0.013	0.152	1.484	0.142
ΔC2–C7 Cobb (°)	-0.004 (-0.045, 0.038)	0.021	-0.017	-0.174	0.863
ΔC2 slope (°)	-0.030 (-0.075, 0.015)	0.023	-0.144	-1.332	0.186
Preoperative VAS	-0.078 (-0.235, 0.079)	0.079	-0.089	-0.984	0.328
Sex (male)	-0.144 (-0.695, 0.407)	0.277	-0.050	-0.518	0.606
Age (years)	0.022 (-0.034, 0.078)	0.028	0.072	0.771	0.443
BMI (kg/m²)	0.030 (-0.068, 0.128)	0.049	0.062	0.607	0.546

Model Summary: *R²* = 0.343, Adjusted *R²* = 0.279, *F*(8, 82) = 5.357, *P* < 0.001

*Abbreviations：B* unstandardized regression coefficient, *CI* confidence interval, *SE* standard error, *β* standardized coefficient

Critically, none of the radiographic Δ values, representing the magnitude of surgical sagittal correction, showed a significant association with the postoperative VAS score (ΔcSVA: B = 0.019, *P* = 0.142; ΔC2–C7 Cobb: B = -0.004, *P* = 0.863; ΔC2 slope: B = -0.030, *P* = 0.186). Furthermore, other covariates including preoperative VAS, age, sex, and BMI were not significantly associated with the outcome in the final model (all *P* > 0.05). To address the potential risk of overfitting due to the inclusion of eight independent variables, we performed a sensitivity analysis using a reduced model that excluded the three Δ radiographic correction parameters and retained only the Goutallier grade, preoperative VAS, age, sex, and BMI. The results remained essentially unchanged: the Goutallier grade remained a significant independent factor associated with the 2‑year VAS score (B = 0.967, 95% CI: 0.649 to 1.285, β = 0.546, *P* < 0.001; model R² = 0.319, Adjusted R² = 0.279), supporting the robustness of our primary finding against overfitting concerns. Variance inflation factor (VIF) values in the reduced model ranged from 1.013 to 1.105, indicating no multicollinearity.

## Discussion

As the classic surgical procedure for treating cervical spondylotic myelopathy, ACDF aims to improve neurological function and restore cervical stability through direct decompression and secure fusion [19]. This study systematically investigated the impact of preoperative paraspinal muscle fat infiltration on 2-year postoperative clinical efficacy in patients with cervical myelopathy who underwent ACDF. The results demonstrated that all patients exhibited significant improvements in radiographic parameters and clinical symptom relief postoperatively. This outcome first supports the core efficacy of ACDF in restoring cervical sagittal plane alignment (such as the T1 slope, C2‒C7 Cobb angle, and C0‒C2 Cobb angle) and neurological function, providing further support for the clinical application of this procedure.

Although all three groups (mild, moderate, and severe fat infiltration) demonstrated significant improvement in sagittal sequences postoperatively, no significant differences were observed in these key imaging parameters between the groups. However, further stratified analysis revealed significant intergroup differences in clinical functional outcomes. Compared with the mild and moderate groups, the severe fat infiltration group demonstrated significantly poorer immediate postoperative VAS scores and worse NDI and VAS scores at 2 years postoperatively. Furthermore, the improvement in the VAS score was markedly lower in the severe group. This phenomenon suggests that the ACDF procedure itself has a high degree of consistency in reconstructing cervical spine structures, effectively restoring the cervical biomechanical balance. Its structural improvement is not significantly influenced by the degree of preoperative muscle‒fat infiltration. Hiyama et al. [20] demonstrated an association between muscle mass and sagittal balance, although surgical intervention generally improved these parameters. However, the functional status of paraspinal muscles—particularly abnormalities caused by fatty infiltration—may play a more critical role in postoperative pain perception and functional adaptation [21]. Notably, although the JOA scores did not significantly differ among the three groups, changes in the NDI and VAS scores demonstrated greater sensitivity. These findings suggest that the influence of fat infiltration on postoperative recovery is more concentrated in the dimensions of patients’ subjective pain and activities of daily living, rather than purely in the restoration of neurological function. Pinter et al. [22] demonstrated the reliability of fat infiltration in assessing cervical muscle functional decline. This finding aligns closely with the previously described characteristics of fat infiltration’s impact on postoperative recovery and provides crucial evidence for understanding the clinical implications of fat infiltration.

Notably, our results demonstrated a clear dose-response relationship: compared to the severe infiltration group, both the mild and moderate groups exhibited superior immediate postoperative pain relief and better long-term functional (NDI) and pain (VAS) outcomes at 2 years, with significantly greater improvement in VAS scores. Although correlational analyses revealed that a higher Goutallier grade was weakly but significantly correlated with greater increases in ΔcSVA and ΔC2 slope (ρ = 0.221 and 0.220, respectively; both *P* < 0.05), no significant intergroup differences were found for these parameters in ANOVA (*P* = 0.110 and 0.105, respectively). The multiple linear regression model showed that the preoperative Goutallier grade was an independent factor associated with the 2-year VAS score, even after controlling for these surgical correction parameters. This finding indicates that the link between severe fat infiltration and suboptimal postoperative recovery is not primarily mediated by a lesser degree of bony alignment correction achievable during surgery. Instead, it underscores that the intrinsic quality and functional impairment of the paraspinal musculature may represent an important independent “soft-tissue determinant” of a patient’s pain experience and functional adaptation following successful structural decompression and fusion.

The core mechanism underlying these clinical outcome differences is closely related to the functional status of the cervical paraspinal muscles (including the multifidus and semispinalis cervicis). As deep spinal extensors, these muscles play an irreplaceable role in the biomechanical stability of the spine [23]. As fatty infiltration progressively increases, the paraspinal muscles in patients with cervical spondylosis may undergo compensatory hypertrophy. However, alterations in the physical properties of muscle tissue—such as reduced contraction efficiency and diminished endurance due to fatty replacement—directly compromise muscle function, potentially affecting proprioception, load distribution, and the compensatory capacity of adjacent segments [24]. This is precisely the key reason why postoperative pain is more pronounced and functional recovery is more delayed in the group with severe fat infiltration. Research has confirmed that the severity of fat infiltration in the multifidus muscle at the C7 segment is significantly associated with poorer improvement in NDI scores following ACDF surgery [9]. The study also revealed that the degree of fatty infiltration in the multifidus and rotator muscles exhibited a significant segment-specific correlation with the severity of central canal stenosis in the cervical spine. Furthermore, in cases of central canal stenosis accompanied by spinal cord compression, more pronounced fatty infiltration is observed in the regions below the stenotic segment. These findings suggest that fatty infiltration not only impacts postoperative recovery but also may be closely associated with the extent of lesion progression [25]. Ma et al. [16] demonstrated that patients requiring cervical spine surgery exhibit significant variations in the degree of paraspinal muscle fat infiltration across different regions and segments, highlighting the limitations of this study, which assessed fat infiltration solely at the C5-6 segment. This finding suggests that evaluating a single segment may not comprehensively reflect the patient’s overall muscle condition.

While fat infiltration has been explored, the clinical significance of the paraspinal muscle cross-sectional area has also emerged as a key research focus. Pinter et al. [22] demonstrated that in most measured paraspinal muscles, quantitative results of the cross-sectional area showed no correlation with increased severity of fat infiltration on the basis of the Goutallier grading system. Recent studies on the lumbar spine have further demonstrated that fat infiltration of the paraspinal muscles is directly associated with low back pain and functional impairment [26, 27], whereas the cross-sectional area of these muscles is unrelated to low back pain or degenerative spinal conditions [28, 29]. This common pattern in spinal research provides valuable insights for cervical spine studies. However, a critical gap remains: while existing research has demonstrated an association between cervical paraspinal muscle fatty degeneration and patient-reported outcomes following ACDF surgery [8], the relationship between the cervical paraspinal muscle cross-sectional area and patient-reported outcomes after ACDF has yet to be explicitly investigated. This leaves an important avenue for future exploration. From a clinical practice perspective, all the aforementioned research findings collectively point to a core recommendation: a comprehensive assessment of a patient’s muscle function is essential in the prevention and treatment of cervical spondylosis. The development of personalized rehabilitation training programs for patients with varying degrees of fat infiltration and segmental muscle status not only enhances muscle strength, reduces fat infiltration, and improves overall paracervical muscle function but also fundamentally slows the progression of muscle degeneration, thereby safeguarding long-term postoperative efficacy.

To evaluate the clinical relevance of our findings, we compared the intergroup differences in VAS and NDI scores with established MCID thresholds. The MCID for VAS-neck pain in ACDF patients ranges from 2.6 to 4.0 points, with a minimum detectable change threshold of 2.6 points [30]. For the NDI, the most commonly used MCID is 7.5 points (out of 50) [31, 32], and a ≥ 30% improvement from baseline (corresponding to an absolute change of approximately 11.3 points) has also been validated [33]. In our cohort, the differences between the severe group and the mild/moderate groups at 2 years were approximately 2.0 points for VAS and 2.6–2.7 points for NDI. While the VAS differences approached the MCID threshold of 2.6 points, the NDI differences fell below the established MCID of 7.5 points. These findings suggest that although severe fat infiltration is associated with statistically significant worse outcomes, the clinical significance of the between-group differences is modest. Nevertheless, the consistent pattern across multiple outcome measures, together with the substantially lower VAS improvement in the severe group (ΔVAS = 1.46 vs. 3.44–3.94 in the other groups), supports the potential prognostic value of preoperative muscle quality assessment. Larger prospective studies are warranted to further define the clinical meaningfulness of these associations.

This study has several limitations. First, its retrospective design may introduce selection bias. Second, the sample size is relatively small, although a sensitivity analysis using a reduced covariate set supported the robustness of our primary findings. Third, fat infiltration was assessed only at the C5–6 segment, which may not fully reflect multilevel fatty degeneration. Fourth, we lacked data on several potential confounders, including symptom duration, smoking status, diabetes mellitus, osteoporosis, T2 high signal intensity of the spinal cord, severity of stenosis, and postoperative rehabilitation, all of which could influence the observed associations between muscle quality and clinical outcomes. Fifth, while the distribution of single- and two-level surgeries was balanced and reported, specific operated levels were not analyzed as covariates due to sample size constraints, and we lacked postoperative imaging to evaluate bony fusion. Sixth, the VAS score was assessed as a general pain score rather than separately for neck and arm pain, which limits our ability to differentiate the effects of fat infiltration on specific pain types. Future studies with separate neck and arm VAS assessments would provide more detailed insights. Future prospective studies with larger sample sizes, multilevel muscle assessments, and detailed clinical and rehabilitation data are needed to validate our findings.

## Conclusion

In summary, this study suggests that anterior cervical discectomy and fusion effectively restores cervical sagittal alignment and alleviates clinical symptoms in older patients with cervical spondylotic myelopathy. However, severe preoperative fat infiltration of the cervical paraspinal muscles is independently associated with inferior postoperative pain relief and long-term functional recovery within this aging population. These findings suggest that preoperative assessment of paraspinal muscle quality may be useful for risk stratification in older patients undergoing ACDF. Future prospective studies are warranted to determine whether interventions targeting paraspinal muscle degeneration can improve postoperative outcomes.

## Data Availability

The data used to support the findings of this study are included within the article.

## Abbreviations

ACDF: Anterior Cervical Discectomy and Fusion
ASDH: Average Segmental Disc Height
CSM: Cervical Spondylotic Myelopathy
cSVA: C2–C7 sagittal vertical axis
JOA: Japanese Orthopaedic Association
NDI: Neck Disability Index
VAS: Visual Analog Scale
ROM: Range of Motion
SSA: Surgical Segmental Angle
VIF: Variance Inflation Factor
ICC: Intraclass Correlation Coefficient
